# Peroxyoxalate Chemiluminescent Reaction as a Tool for Elimination of Tumour Cells Under Oxidative Stress

**DOI:** 10.1038/s41598-017-03527-w

**Published:** 2017-06-13

**Authors:** Andrey V. Romanyuk, Irina D. Grozdova, Alexander A. Ezhov, Nickolay S. Melik-Nubarov

**Affiliations:** 1 M.V. Lomonosov Moscow State University, Department of Chemistry, GSP-1, Leninskie gory 1, build. 3, Moscow, 119991 Russia; 2 M.V. Lomonosov Moscow State University, Department of Physics, GSP-1, Leninskie gory 1, build. 2, Moscow, 119991 Russia

## Abstract

The overproduction of hydrogen peroxide is an inherent feature of some tumour cells and inflamed tissues. We took advantage of this peculiarity to eliminate cells using chemiluminescent peroxyoxalate reaction. We designed dispersions containing polyoxalate and tetramethylhematoporhyrin (TMHP) in dimethylphthalate droplets stabilized with Pluronic L64. The porphyrin plays the dual role. On the one hand, it serves as an activator of the peroxyoxalate reaction of polyoxalate with intracellular hydrogen peroxide and experiences excitation as a result of the reaction. The light emitted in the reaction in the model system without cells was used to optimize the dispersion’s composition. On the other hand, TMHP acts as a photosensitizer (PS) causing cell damage. The formation of singlet oxygen led to cell elimination if the dispersions were used in combination with inducers of oxidative stress: hydrogen peroxide, paraquat, antitumour drug doxorubicin, or a nutritional additive menadione. The PS-induced cytotoxicity correlated with the level of intracellular ROS. The developed approach targeted to endogenous ROS is orthogonal to the classical chemotherapy and can be applied to increase its efficiency.

## Introduction

Photodynamic therapy (PDT) implies photochemical interaction of three components: light, photosensitizer (PS), and oxygen. Molecules of a PS, localizing into or in proximity to cells and exposed to external light source, turn into excited state and then transfer energy to ambient molecules, including molecular oxygen. This two-step process leads to generation of singlet oxygen, an extremely strong oxidizing agent that destroys cellular components thus causing toxic effects^1^. Excitation of PS molecule is the key step, which defines the effectiveness of the approach.

Despite significant progress in technical development of PDT, it is not free from limitations. One of them is the opacity of tissues that interferes with application of PDT for treatment of visceral and metastatic tumours^2–5^. In order to overcome this limitation, chemiluminescent reactions as a source of light have been proposed. Application of luciferase catalyzed oxidation of luciferin resulted in the development of “molecular flashlight” sufficient for PS excitation without external light source^6, 7^.

**Figure 1:**
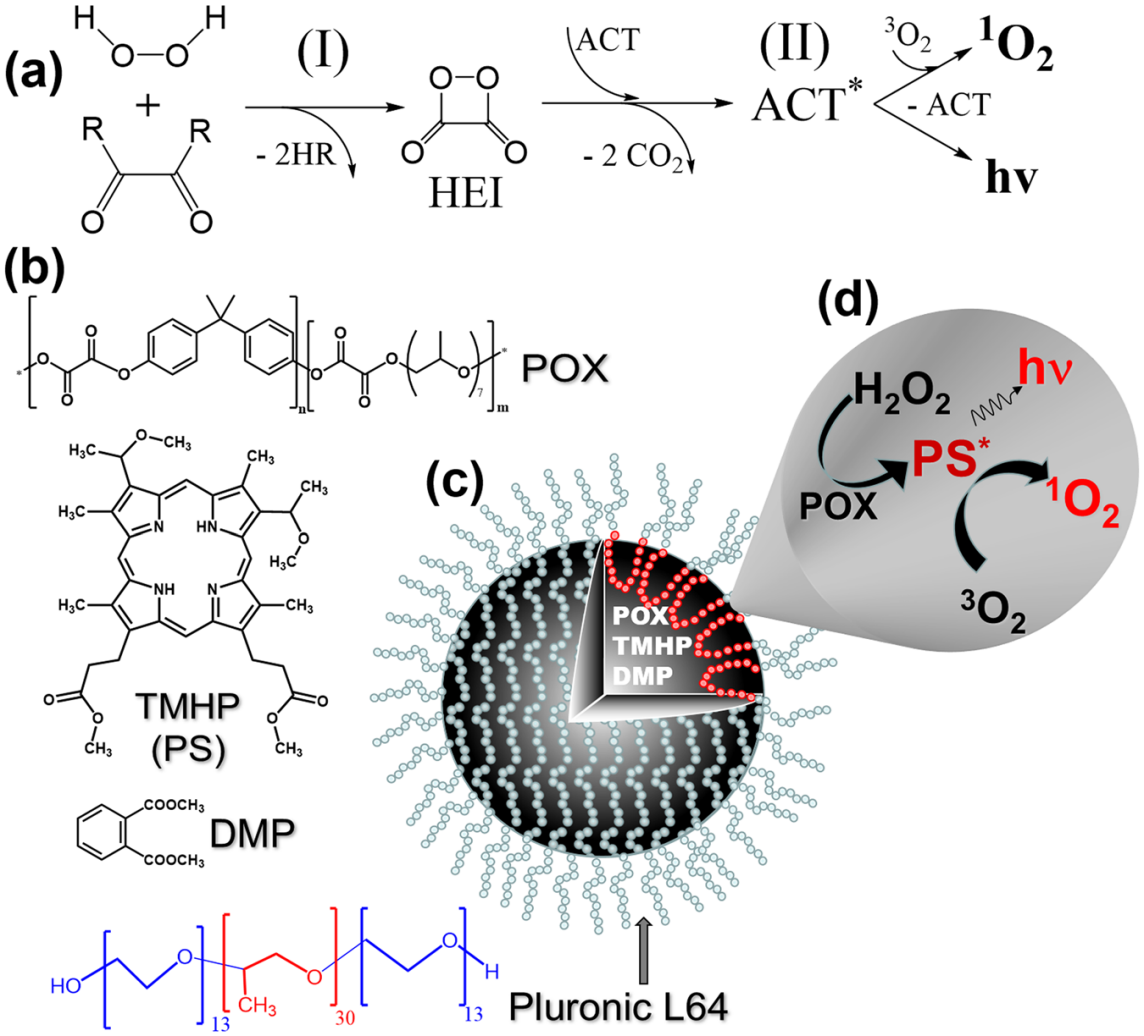
Scheme of the designed approach. (**a**) Scheme of peroxyoxalate chemiluminescent reaction. R – electron-withdrawing group. (**b**) Structures of Polyoxalate (**POX**), tetramethylhematoporphyrin IX (**TMHP**), dimethylphthalate (**DMP**), and Pluronic L64; (**c**) Sketch of the dispersion particles containing POX and TMHP in droplets of DMP stabilized with Pluronic L64; (**d**) PO-CL reaction with endogenous H_2_O_2_ results in light emission and production of singlet oxygen capable to eliminate tumour cells.

Phillip *et al*. were the first who employed peroxyoxalate chemiluminescent (**PO-CL**) reaction as an internal light source^8, 9^. The reaction proceeds between active derivatives of oxalic acid and hydrogen peroxide (Fig. 1a). The first step of the reaction is the nucleophilic attack of an oxalate by a molecule of peroxide-anion which results in formation of high-energy intermediate (**HEI**) 1,2-dioxetanedione (Fig. 1a, I). In the presence of activators (**ACT**) (different polyaromatic compounds), 1,2-dioxetanedione is able to transfer excess energy to these molecules while experiencing decomposition into two CO_2_ molecules (Fig. 1a, II)^10–12^.

Phillip *et al*. used PO-CL reaction for elimination of tumour cells^8, 9^. Transplanted tumours were treated with a mixture of an active water-soluble oxamide, 1% solution of hydrogen peroxide and disulforubrene served as an ACT. Chemiluminescence emitted by rubrene was absorbed by PS preliminary accumulated in the tumour thus creating conditions for intracellular generation of singlet oxygen.

In this work the exogeneous hydrogen peroxide was used to trigger the chemiluminescent reaction. However large amounts of endogenous peroxide are often found in malignant cells and inflamed tissues^13–15^. This peculiarity provides the possibility to run PO-CL reaction without addition of exogeneous peroxide and provoked a number of approaches to visualization and elimination of cells with increased ROS production. The first chemiluminescent system triggered by endogenous peroxide was presented by Laptev *et al*.^16^. In this work, oxidation of luminol with endogeneous H_2_O_2_ resulted in light emission. The emitted chemiluminescence was absorbed by the PS attached to the transferrin molecule thus inducing selective elimination of cells with elevated amounts of transferrin receptors.

Further, Lee *et al*. designed polyoxalate-containing nanoparticles responsive to endogenous hydrogen peroxide for *in vivo* imaging of inflamed tissues in mice^17^. Later, peroxyoxalate chemiluminescent systems combined with the effect of aggregation-enhanced fluorescence^18^ and semiconducting polymers^19^ were applied for detection of hydrogen peroxide associated with lipopolysaccharide-induced inflammation.

The goal of the present study was to construct polyoxalate-containing dispersions capable of elimination of tumour cells through PO-CL reaction with endogenous hydrogen peroxide in the presence of tetramethylhematoporphyrin (TMHP) (Fig. 1d). The latter participated as an activator in PO-CL reaction^20^ as well as an effective PS for singlet oxygen generation, thus avoiding a superfluous step of ACT to PS energy transfer. POX and TMHP were formulated into dispersion droplets of dimethyl phthalate stabilized with Pluronic L64 as a surfactant (Fig. 1c). Cytotoxicity of these formulations toward human breast adenocarcinoma MCF-7/ADR cells without external light illumination was studied.

## Results

### Synthesis of polymeric oxalate

Polymeric oxalates have been reported to be comparatively resistant to hydrolysis and therefore can be used in PO-CL reaction in aqueous environment^17, 21^. POX (Fig. 1b) was synthesized through polycondensation of oxalyl chloride, bisphenol A and oligo(propylene glycol) (see Supplementary Fig. S1).

The polymer had a wide molecular weight distribution (see Supplementary Fig. S2). Molecular weight characteristics were estimated from calibration using polystyrene standards. M_w_, M_n_ and PDI values were found to be 4400, 1700, and 2.6, respectively. POX composition was determined with ^1^H-NMR and the molar ratio of monomers bisphenol A/oligopropylene oxide was found to be 0.85:0.15 (see Supplementary Fig. S3).

### Preparation of polyoxalate dispersions

Since aromatic oxalates are susceptible to hydrolytic degradation, we endeavoured to diminish contact of POX with water in the course of dispergating procedure. To this end, POX was first dissolved in aprotic solvents. DMP and THF were used as the best solvents for POX among other tested. The solution was mixed with Pluronic L64 and then about 100-fold volume of aqueous buffer (PBS) was injected into the vial under intensive shaking at 37 °C resulting in spontaneous formation of dispersions. The surfactant forms a hydrophobic core and a hydrophilic corona which stabilizes the particles. Herein, the concentration of the dispersions is expressed in mg/mL of Pluronic L64 at an indicated weight ratio of other components.

**Figure 2:**
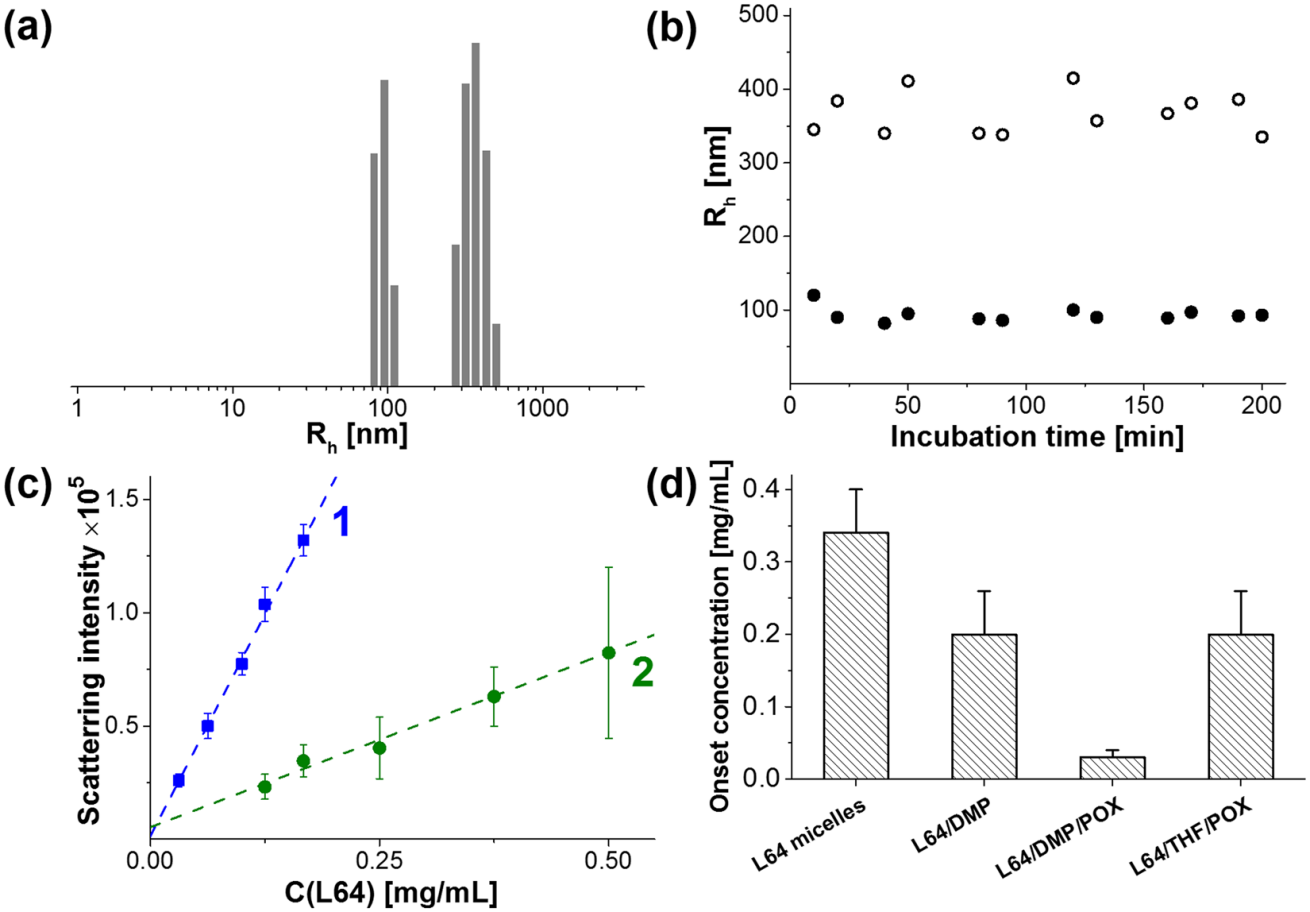
Characterization of the POX-containing dispersions. (**a**) Size distribution of the dispersion prepared from POX solution in DMP. (**b**) Stability of hydrodynamic radii of two fractions of L64/POX/DMP dispersions over time (L64/DMP/POX = 1:2.7:0.3 by mass, Pluronic L64 concentration 0.25 mg/mL, PBS, 37 °C). (**c**) Concentration dependence of the mean intensity of light scattered at an angle 90 °C from the dispersion (1) L64/DMP/POX (1:2.7:0.3 by mass) and (2) L64/THF/POX (1:2.7:0.3 by mass) in PBS, 37 °C. Scattering intensity was measured in triplicates at least in 2 independent experiments. (**d**) Critical concentrations corresponding to the onset of hydrophobic phase probed by an increase in DPH fluorescence.

The dynamic light scattering of the dispersions prepared from POX solutions in DMP and THF revealed two types of particles in both formulations. DMP led to formation of particles with the average hydrodynamic radii (R_h_) about 105 ± 25 nm and 400 ± 100 nm (Fig. 2a), which remained practically unchanged during several hours (Fig. 2b). When THF was used instead of DMP, the particles were considerably smaller (R_h_ about 23 ± 9 and 100 ± 30 nm).

Dilution of both dispersions resulted in a linear decrease in scattering intensity within the concentration range from 0.03 to 0.25 mg/mL of Pluronic L64 at a fixed Pluronic/POX/DMP weight ratio (1:0.3:2.7) (Fig. 2c, curve 1). It means that the particles retained even after 10-fold dilution of the dispersion. Similar results were obtained for the dispersions prepared from POX solution in THF (Fig. 2c, curve 2). Scattering intensity in this case was considerably less due to lower size of the particles.

The substantial difference between the dispersions prepared from POX solutions in THF and DMP was further confirmed by solubilization of the hydrophobic probe 1,6-diphenyl-1,3,5-hexatriene (**DPH**). This technique is based on the increase in fluorescence of DPH upon its partitioning into hydrophobic microphase^22^ (Fig. S4 in Supplementary Information). High sensitivity of this approach affords an opportunity to determine the critical concentration of the dispersions of various compositions corresponding to the onset of a hydrophobic microphase.

Formation of a hydrophobic core in the solutions of Pluronic L64 was observed at concentrations above 0.35 mg/mL in complete agreement with the previously published data^23^. Pluronic L64/DMP or L64/DMP/POX dispersions formed a hydrophobic microphase at 1.5 and 10-fold less concentrations (Fig. 2d), indicating that the solubilization of DMP and even more POX/DMP in Pluronic micelles stabilized the particles upon their dilution. In the latter case the critical concentration was as low as 0.03 mg/mL in full agreement with light scattering data (cf. Fig. 2d, curve 1 and 3c). In contrast, POX solution in THF caused only marginal effect on the critical concentration of the dispersions (Fig. 2d). Neither DMP, nor POX formed a hydrophobic phase in the absence of Pluronic L64.

These results show that THF used as a solvent for POX most probably undergoes partitioning into the continuous phase resulting in the formation of the dispersion particles made only of Pluronic and POX. On the contrary, if the dispersions were prepared from POX solution in DMP, the hydrophobic core contained not only Pluronic and POX but also DMP.

### Efficiency of PO-CL reaction in buffer solution as a function of dispersion’s composition

**Figure 3:**
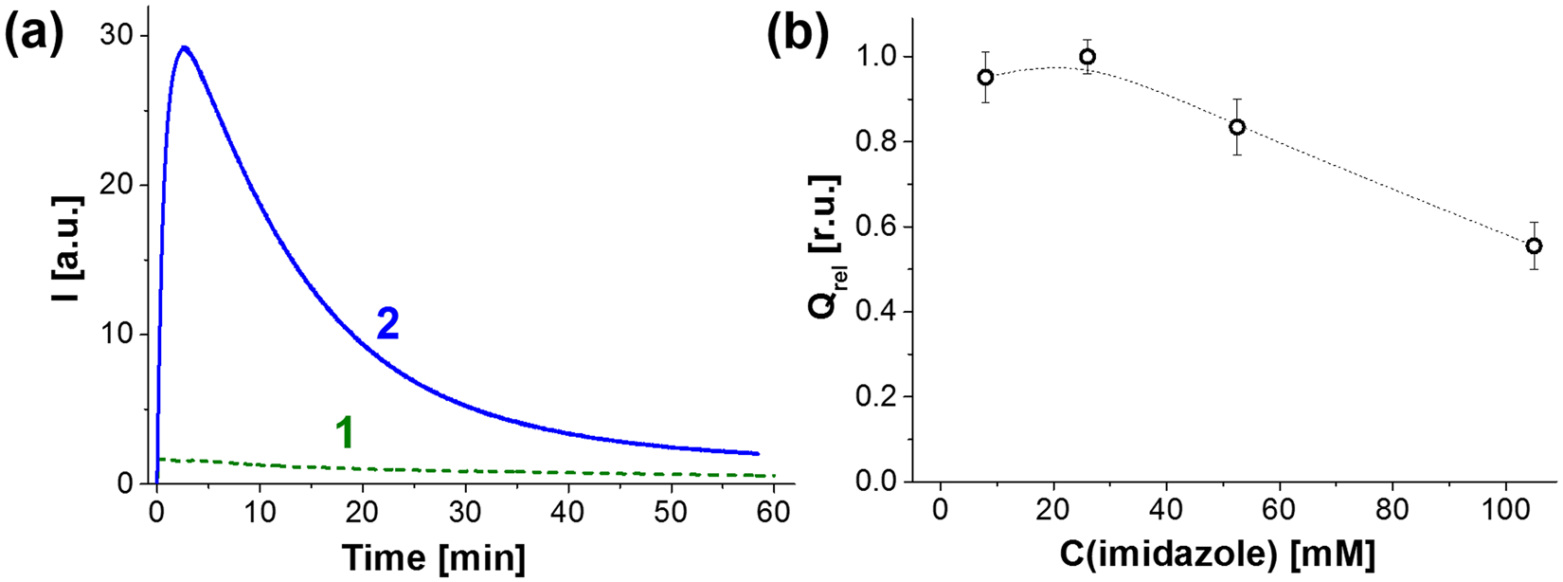
Influence of imidazole on PO-CL reaction. (**a**) The kinetics of light emission in the absence of imidazole (1) and in the presence of 26 mM imidazole (2). (**b**) Dependence of the relative integral intensity of chemiluminescence of the dispersions on the concentration of added imidazole. C(L64) = 1.0 mg/mL, C(DMP) = 2.66 mg/mL, C(POX) = 0.30 mg/mL, C(TMHP) = 7.6 μM, C(H_2_O_2_) = 15.0 mM. The integral intensities were normalized to the value obtained at 26 mM of imidazole (Q_rel_ = Q/Q_max_).

PO-CL reaction between POX and hydrogen peroxide was performed in the presence of TMHP which participated as an activator^20^ and an effective PS^1^, combining both properties. The rate of the chemiluminescence reaction was very slow (Fig. 3a, curve 1). It was accelerated by more than an order of magnitude by addition of imidazole, an established catalyst of this reaction^24^ (Fig. 3a, curve 2). Previously^25–28^, it was demonstrated that it possesses both base and nucleophilic catalytic activity and it accelerates the hydrogen peroxide attack on oxalate moiety and the formation of high-energy intermediates which is the rate-determining step of the whole process (Fig. 1a). This resulted in faster decay of chemiluminescence intensity and allowed reducing the time of light measurement to more convenient intervals (about half an hour).

Concentrations of imidazole below 30 mM did not affect the integral intensity of light (Q) emitted during the chemiluminescent reaction, while further increase in the concentration of the catalyst caused a slight decrease in chemiluminescence efficiency (Fig. 3b) in full agreement with the previous reports^24, 29^. So, we performed all further experiments in the presence of 26 mM imidazole.

Concentrations of imidazole below 30 mM did not affect the integral intensity of light (Q) emitted during the chemiluminescent reaction, while further increase in the concentration of the catalyst caused a slight decrease in chemiluminescence efficiency (Fig. 3b) in full agreement with the previous reports^24, 29^. So, we performed all further experiments in the presence of 26 mM imidazole.

The integral intensity of light emitted by DMP-containing dispersions was two orders of magnitude higher than that obtained with THF, indicating a striking difference between the arrangement of components in L64/DMP/POX and L64/THF/POX dispersions.

L64/DMP/POX dispersions capable of chemiluminescence generation in PO-CL reaction will be referred to as chemiluminescence dispersions (**CLD**). Their composition was optimized to achieve the maximum efficiency of PO-CL reaction.

**Figure 4:**
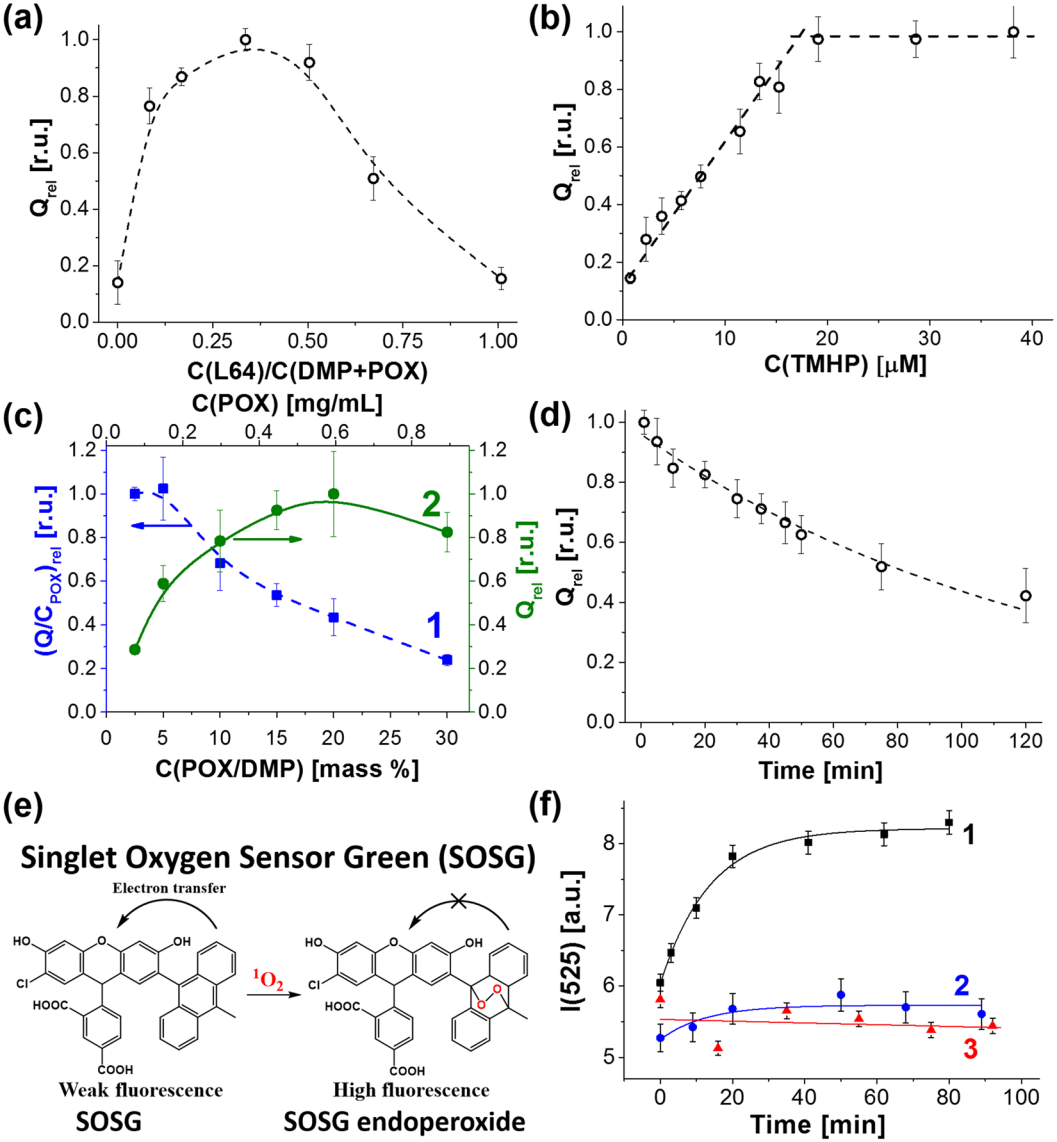
Relative integral intensity of PO-CL reaction (Q_rel_ = Q/Q_max_) as a function of the composition of the dispersions and evidence for singlet oxygen generation in PO-CL reaction in cell-free system. (**a**) Variation of Pluronic L64 content expressed as its weight ratio toward the organic phase (DMP+POX). C(DMP) = 2.7 mg/mL, C(POX) = 0.3 mg/mL, C(TMHP) = 7.6 μM; (**b**) Variation in TMHP concentration. C(L64) = 1.0 mg/mL, C(DMP) = 2.7 mg/mL, C(POX) = 0.3 mg/mL. (**c**) Variation in POX content: (1) normalized PO-CL efficiency per POX amount (left axis, (Q/C_POX_)_rel_ = (Q/C_POX_)/(Q/C_POX_)_max_) and (2) the relative integral intensity (right axis) on the overall POX concentration (top axis) and POX fraction in DMP solution (bottom axis). C(L64) = 1.0 mg/mL, C(DMP+POX) = 3.0 mg/mL, C(TMHP) = 7.6 μM; (**d**) The kinetics of hydrolysis of POX in the dispersions of L64/DMP/POX (1:2.7:0.3 weight ratio). In all experiments C(imidazole) = 26 mM, C(H_2_O_2_) = 15.0 mM. (**e**) Structure of Singlet Oxygen Sensor Green fluorescent probe and the reaction of its oxidation by singlet oxygen. (**f**) Kinetics of SOSG oxidation in the presence of 30 µM H_2_O_2_ and complete CLD (1), the dispersion without TMHP (2), or POX (3).

The increase in the concentration of Pluronic L64 at a constant concentration of the hydrophobic components (2.7 mg/mL of DMP and 0.3 mg/mL of POX) led to a steep growth in integral intensity of chemiluminescence that reached a smooth maximum at the weight fraction of Pluronic being 0.34 (Fig. 4a). According to the classical concept of dispersion stability^30^ this amount of Pluronic corresponds to its minimum sufficient for coating the total surface of the particles. The PO-CL efficiency slowly receded at higher content of Pluronic.

The chemiluminescence intensity increased linearly with the concentration of the activator up to 20 μM TMHP (Fig. 4b) in complete agreement with the mechanism of PO-CL reaction (Fig. 1a). However, above this value the chemiluminescence intensity levelled off obviously due to the self-quenching of porphyrin luminescence.

Variation of POX concentration in DMP solution at a fixed concentration of TMHP was found to be of primary importance for the efficiency of PO-CL reaction. The integral intensity normalized to the amount of POX decreased with the growth of POX concentration in DMP (Fig. 4c, curve 1). Low POX concentrations (2.5 and 5.0 mass % in DMP) form the plateau on the plot and provide the highest chemiluminescence yield. However, these two points are characterized by relatively low absolute intensities (Fig. 4c, curve 2, right axis). At the same time, the dispersions prepared from 10% and 30% of POX in DMP produced nearly similar integral intensities of chemiluminescence (Fig. 4c, curve 2). Therefore, we accepted 10 mass % to be the optimum POX content in DMP since it provides high integral intensity (Q, right axis) as well as a suitable chemiluminescence efficiency (Q/C_POX_).

Based on these results, we determined the optimum L64/DMP/POX/TMHP weight ratio as equal to 1:2.7:0.3:0.05.

Stability of POX toward hydrolysis in the dispersions prepared from 10 mass % of POX in DMP in the presence of 1.0 mg/mL of Pluronic L64 was evaluated by incubation of the dispersions in aqueous buffer at 37 °C during various time intervals before injection of hydrogen peroxide and imidazole. Half-life period of POX in the dispersions was found to be about 1.4 h (Fig. 4d) that is appropriate for cell culture experiments. Further we studied the capacity of CLD to generate singlet oxygen due to PO-CL reaction.

### Generation of singlet oxygen during PO-CL reaction in the cell-free system

To obtain experimental evidence for singlet oxygen generation in PO-CL reaction, we used the commercially available probe Singlet Oxygen Sensor Green (SOSG, Fig. 4e). Fluorescent properties of fluorescein are suppressed in this molecule due to intramolecular electron transfer. Oxidation of anthracene moiety in SOSG by singlet oxygen destroys the conjugation system and restores bright fluorescence of chlorofluorescein fragment.

The manufacturer of SOSG reports extremely high responsiveness of SOSG to singlet oxygen^31^. Unexpectedly, we observed a fast oxidation of SOSG in the presence of 20 mM H_2_O_2_ without a PS (see Supplementary Fig. S5a), the reaction being occurred until exhaustion of all SOSG. However, the oxidation rate decreased in line with H_2_O_2_ concentration and became negligible at 100 μM and 30 µM H_2_O_2_ (see Supplementary Fig. S5a). This fact clearly showed that SOSG could be used for monitoring ^1^O_2_ formation during PO-CL reaction in the presence of low H_2_O_2_ concentrations.

In the presence of complete CLD, the kinetics of oxidation of SOSG in the presence of 30 µM of H_2_O_2_ obeyed the monoexponential law with characteristic time about 20 min that corresponded to the kinetics of PO-CL reaction (Fig. 4f, curve 1). Further incubation of the reaction mixture after completion of PO-CL reaction did not result in an additional oxidation of SOSG. It means that SOSG oxidation in this case occurred due to the reaction with singlet oxygen generated by TMHP during PO-CL reaction, rather than direct oxidation by H_2_O_2_ or by molecular (atmospheric) oxygen. Importantly, the omission of either POX or TMHP in CLD formulation completely prevented SOSG from oxidation at low concentrations of H_2_O_2_ (Fig. 4f, curves 2 and 3).

It is known, that porphyrins triplet state lifetime is considerably enhanced in deuterated solvents. We observed that the replacement of water by D_2_O resulted in a two-fold increase in the amount of SOSG endoperoxide produced during PO-CL reaction in the presence of the complete CLD (see Supplementary Fig. S5b). This result gave additional argument in favour formation of singlet oxygen during PO-CL reaction in the cell-free system.

### Interaction of chemiluminescent dispersions with cells

#### Cytotoxicity of dispersions

**Figure 5:**
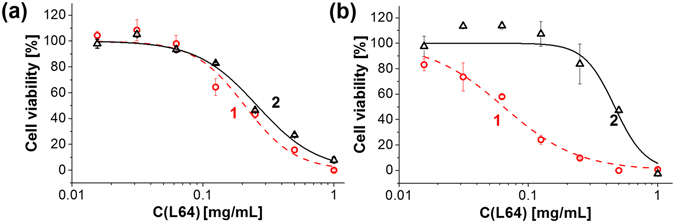
Interaction of CLD with MCF-7/ADR cells. Dose-response survival curves of MCF-7/ADR cells either untreated (**a**), or pretreated with 200 μM H_2_O_2_ (**b**) in the presence of complete CLD (1) and reference dispersion deprived of TMHP (2).

CLD formulations were tested on multidrug resistant human breast adenocarcinoma MCF-7/ADR cells as an example of refractory cell line. The cytotoxicity of the CLD formulations (Fig. 5a, curve 1) was compared to that of the reference dispersion deprived of TMHP (Fig. 5a, curve 2). Their IC50 values were nearly similar within the experimental error (0.21 ± 0.03 and 0.26 ± 0.02 mg/mL respectively). We suggested that inability of CLD to eliminate cells was due to insufficient concentration of endogenous hydrogen peroxide necessary for PO-CL reaction.

To increase the content of endogenous ROS, MCF-7/ADR cells were subjected to oxidative stress induced by hydrogen peroxide^32, 33^ at a non-toxic concentration (about 200 µM) (Fig. S6 in Supplementary Information shows hydrogen peroxide cytotoxicity dose-response curve).

Thus pretreated cells exhibited a considerable difference between IC_50_ values of CLD (Fig. 5b, curve 1) and the reference dispersion (Fig. 5b, curve 2). The $I{C}_{50}^{REF}/I{C}_{50}^{CLD}$ ratio used as a measure of CLD cytotoxicity varied from 3 to 5 in independent experiments.

The increase in CLD cytotoxicity could result from the interaction of CLD either with endogenous H_2_O_2_ or the traces of extracellular H_2_O_2_ used for the treatment of cells. To evaluate the extracellular concentration of H_2_O_2_ we applied the well-known approach based on the oxidation of non-fluorescent dihydro-dichlorofluorescein diacetate (H_2_DCF-DA) by intracellular ROS into fluorescent dichlorofluoresceine (**DCF**)^34^. It is based on the fact that H_2_DCF-DA itself cannot be oxidized, but its deacetylation within cells makes it susceptible to oxidation by hydroxyl radicals or superoxide anion-radicals (see Supplementary Fig. S7a)^35^. These ROS are generated from H_2_O_2_ by peroxidases. The extracellular concentration of H_2_O_2_ was measured by means of the approach originally developed by Rota *et al*.^35^ and further used in a number of papers^36, 37^. It consists in the preliminary hydrolysis of H_2_DCF-DA to the deacetylated non-fluorescent form H_2_DCF and its further oxidation with H_2_O_2_ to highly fluorescent DCF in the presence of horseradish peroxidase, the detection limit of this method being about 50 nM of H_2_O_2_ (see Supplementary Fig. S7b). Application of this approach showed that after treatment of cells with H_2_O_2_ their exposure to complete medium results in the decay of peroxide concentration in the medium nearly to zero due to the presence of serum catalase (see Supplementary Fig. S7c)^38^.

At the same time, the level of intracellular fluorescence of DCF in the same samples increased after 1 h incubation of the cells in the presence of 200 µM H_2_O_2_ and remained constant during at least 1.5 h incubation in the serum-containing medium (see Supplementary Fig. S7d).

Thus, a significant PS-mediated cytotoxicity was observed in the cells under oxidative stress. Of special note is that the intracellular PO-CL reaction occurred with hydrogen peroxide biosynthesized in response to stimulation.

### Chemiluminescence in MCF-7/ADR cells under oxidative stress conditions

**Figure 6:**
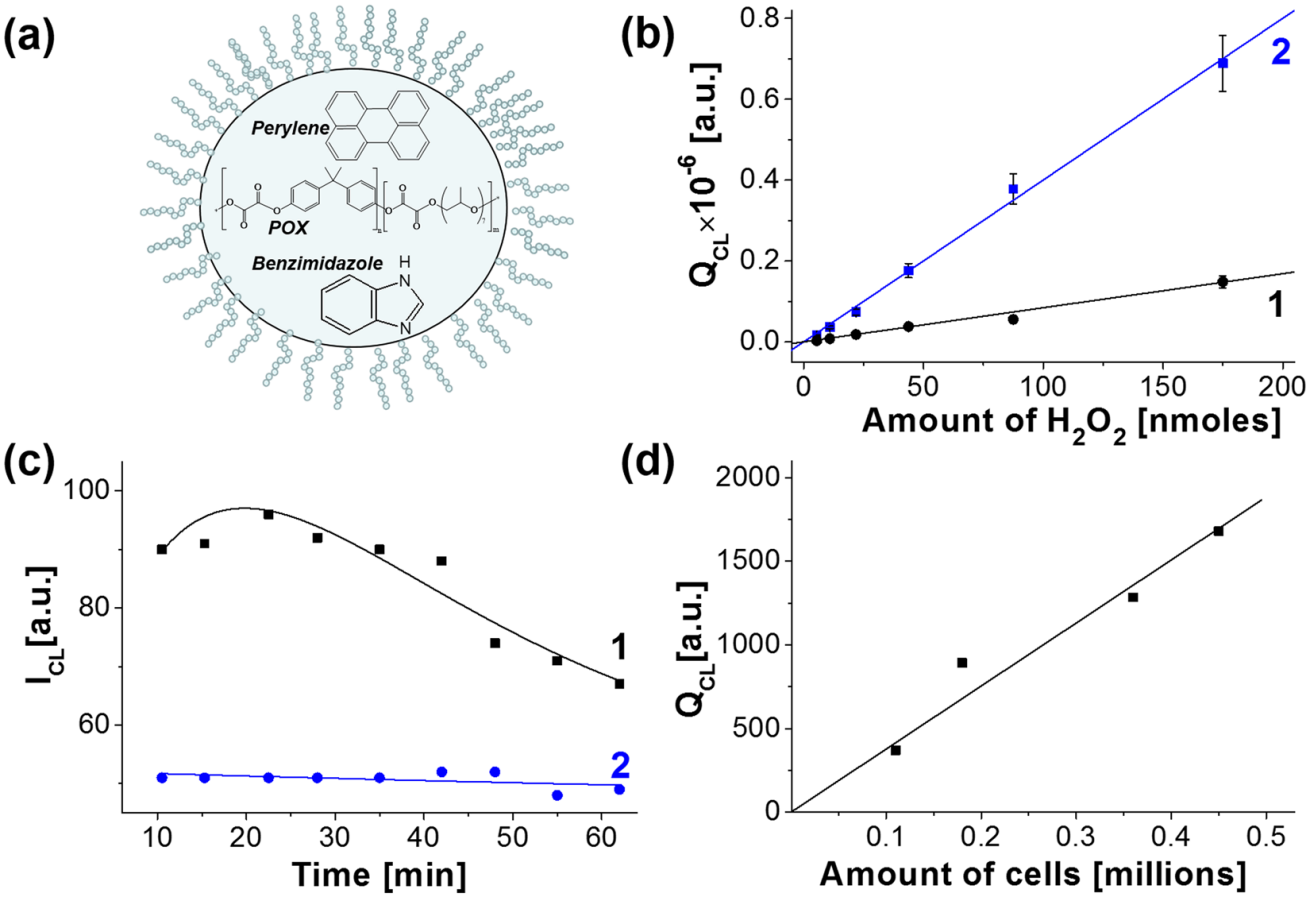
PO-CL reaction in cells under oxidative stress conditions. (**a**) Scheme of the modified CLD formulation designed for the enhancement of chemiluminescence efficiency. (**b**) Dependences of chemiluminescence on the amount of H_2_O_2_ for TMHP- (1) and perylene- (2) based CLD. Concentrations of the activators were 0.48 mM in both cases. TMHP-based dispersion contained 26 mM imidazole, while perylene-based dispersion contained 1 mM benzimidazole. (**c**) Kinetics of light emission by 0.45 millions of MCF-7/ADR cells after addition of 0.2 mL of complete CLD containing 2.1 mM POX repeat units, 0.48 mM perylene and 1 mM benzimidazole (1) and a control formulation without perylene (2). The dispersions were prepared in DMEM supplemented with 10 mM HEPES, pH 7.4 without serum, bicarbonate and Phenol Red, 37 °С. The data were obtained using plate luminometer. (**d**) The dependence of the integral light emitted during PO-CL reaction in the cells on their amount in the sample.

Use of CLD formulations did not allow detecting light emission in cells using LCSM technique. Obviously this was due to special features of hematoporphyrin, which is supposed to be a very efficient photosensitizer (quantum yield of singlet oxygen generation is 0.74^39^), but rather poor fluorophore (fluorescence quantum yield in mitochondria was found to be 0.11–0.13^40^). To enhance the rate of light emission we modified CLD composition. TMHP was replaced for perylene (Fig. 6a) that does not exhibit photosensitizing activity, but manifests extremely high fluorescence quantum yield (about 0.92)^41^. This fluorophore emits blue light which is most effectively detected by the majority of photomultipliers. Another apparent factor capable of increasing the rate of light emission in PO-CL reaction is addition of a nucleophilic catalyst. Imidazole effectively used in cell-free system (Fig. 3) is highly toxic for cells in culture. In addition, it poorly accumulates in the cells owing its hydrophilicity. Instead, we used benzimidazole (Fig. 6a) that readily partitions into the organic phase of CLD and therefore can be used at much lower concentrations. So, the modified CLD formulation contained 1.0 mg/ml Pluronic L64, 2.5 mg/ml DMP, 2.0 mM POX, 0.4 mM perylene and 1 mM benzimidazole.

The designed dispersion was considerably more sensitive to H_2_O_2_ than that based on TMHP/imidazole (Fig. 6b). Addition of CLD containing perylene and benzimidazole to the monolayer of cells under oxidative stress conditions (about 0.45 millions per sample) resulted in a detectable emission of light (Fig. 6c). These result confirmed that PO-CL reaction can be triggered by intracellular hydrogen peroxide.

Intensity of light depended on cell density. The integral of emitted light decreased with diminution in the amount of cells (Fig. 6d) confirming that hydrogen peroxide in the sample was originated from the cells and not from the contamination with H_2_O_2_ used for oxidative stress stimulation.

The linear character of this dependence gave an opportunity to evaluate the amount of hydrogen peroxide in MCF-7/ADR cells in oxidative stress state. The ratio of the slopes of lines in Fig. 6d and line 2 in Fig. 6b gave about 0.9 nmoles per million of cells. Noteworthy, this concentration was detected in the cells stimulated with the maximum tolerable concentration of H_2_O_2_.

### The effect of CLD composition on the PS-mediated cytotoxicity

Thus, we have found that the CLD composition optimized by means of chemiluminescence measurements in cell-free systems appeared to be optimum to cause PS-mediated cytotoxicity *in vitro* (see Fig. S8 and the corresponding explanations in Supplementary Information online). This fact implies that the CLD cytotoxicity was mainly determined by PO-CL reaction resulting in production of toxic species.

### Accumulation of the dispersions in the cells and generation of singlet oxygen due to PO-CL reaction

**Figure 7:**
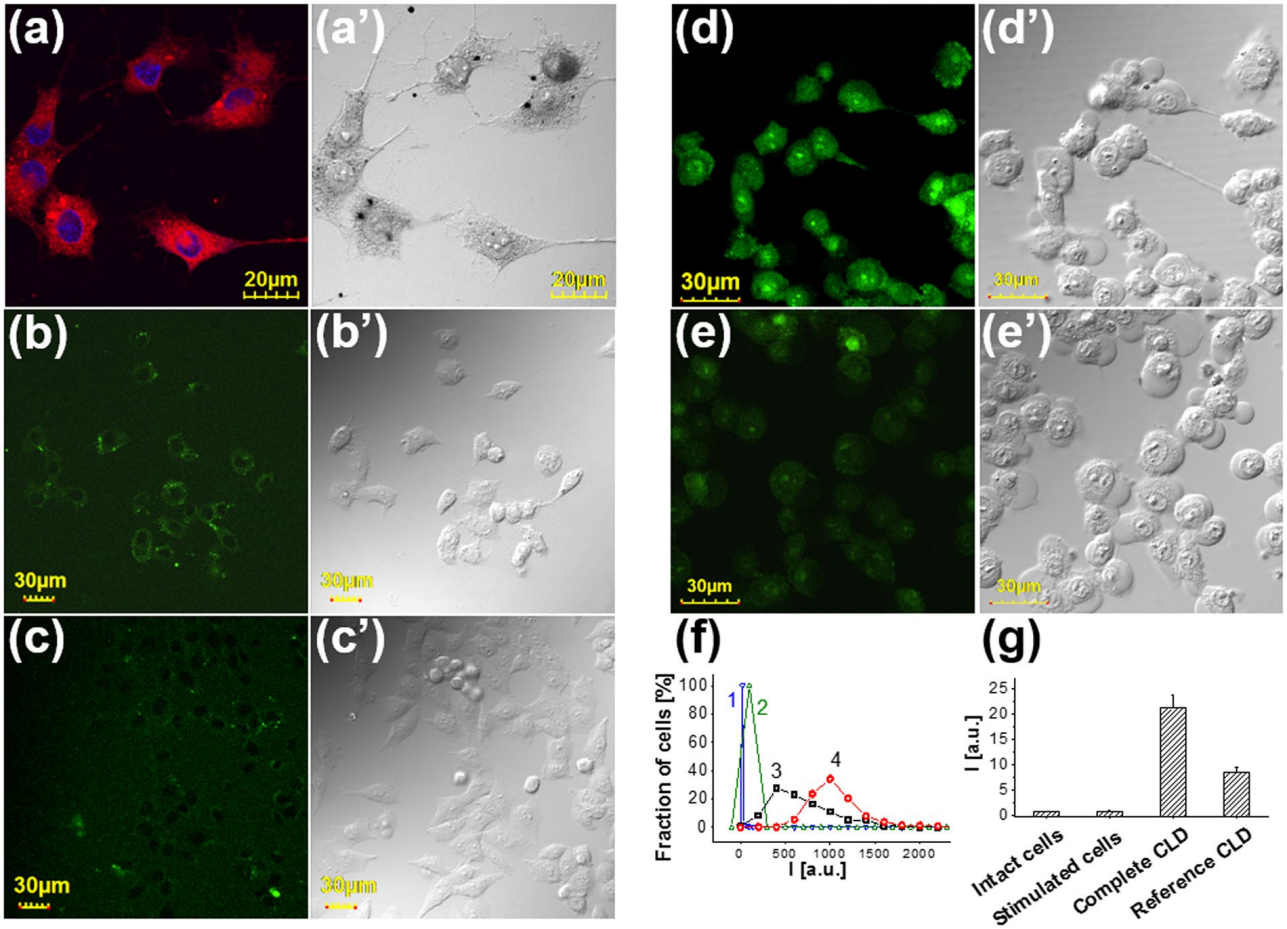
Intracellular localization of TMHP and oxidized SOSG in the cells treated with CLD. (**a**) LCSM image of the cells treated with complete CLD in the serum-free culture medium for 1 h at 37 °C and fixed with 4% formaldehyde in PBS. Nuclei were stained with 0.3 µM DAPI. The left panel shows merged blue and red channels, the right panel shows differential interference contrast image. (**b**–**g**) Evidence for singlet oxygen generation due to PO-CL reaction in the cells. Confocal images of (**b**) intact cells, (**c**) the cells in the oxidative stress state, (**d**) similarly prepared cells treated with the complete and (**e**) reference CLD without TMHP. All samples were stained with 10 mM of SOSG (**f**) Distributions of the cells by the intensity of their fluorescence calculated from the analysis of at least 10 different images containing about 300 cells. 1 – intact cells, 2 – the cells under oxidative stress conditions, 3 – the cells prepared as in the sample (2) and treated with the complete CLD or 4 – with the reference dispersion without TMHP. (**g**) Measurement of SOSG endoperoxide fluorescence in the similarly prepared samples using plate fluorometer. Each sample contained about 0.2 millions of cells.

Cells treatment with CLD resulted in an uptake of the TMHP-containing particles and their distribution all over the cytoplasm (red fluorescence) except for nuclei (blue fluorescence) (Fig. 7a). To obtain evidence for singlet oxygen generation in the cells due to PO-CL reaction we used SOSG. This probe is considered by the manufacturer to be cell-impermeable. However, it was shown that in the absence of serum it is accumulated in cells^42^ and can be oxidized by singlet oxygen produced by different photosensitizers^43–46^. Addition of SOSG to the intact cells (Fig. 7b) or the cells stimulated with H_2_O_2_ (Fig. 7c) resulted in a subtle staining of cytosol. However, addition of complete CLD with SOSG to the stimulated cells resulted in a considerable staining of all cellular compartments including nuclei (Fig. 7d). In contrast, the CLD without TMHP caused much lesser staining than complete CLD (Fig. 7e). However, some cells in this sample were considerably brighter than untreated cells (Fig. 7b and c).

Distributions of cells by the fluorescence intensity in the samples (a-d) show that the mean fluorescence of the cells in the sample treated with CLD is twice of that treated with the dispersion without TMHP (Fig. 7f). Similar results were obtained in the independent experiment, where the cell monolayers were treated with different compositions and SOSG-endoperoxide fluorescence was measured using plate fluorometer (Fig. 7g).

It seems probable that a considerable amount (about 15%) of brightly stained cells in the sample treated with the control dispersion (Fig. 7e) is caused by the ability of SOSG to serve not only as a photosensitizer that has been shown previously^46^, but also as an activator of PO-CL reaction. This feature is peculiar for condensed polyarenes including anthracene and diphenylanthracene^47^ and therefore the ability of anthracene part of SOSG to be an activator for peroxyoxalate chemiluminescence appears to be expectable. This may be a reason for the comparatively high level of SOSG endoperoxide fluorescence in the samples with omitted TMHP.

So, the obtained results give evidence for the formation of singlet oxygen due to PO-CL reaction both in aqueous solution and in cells

### Level of oxidative stress and PS-mediated CLD cytotoxicity

**Figure 8:**
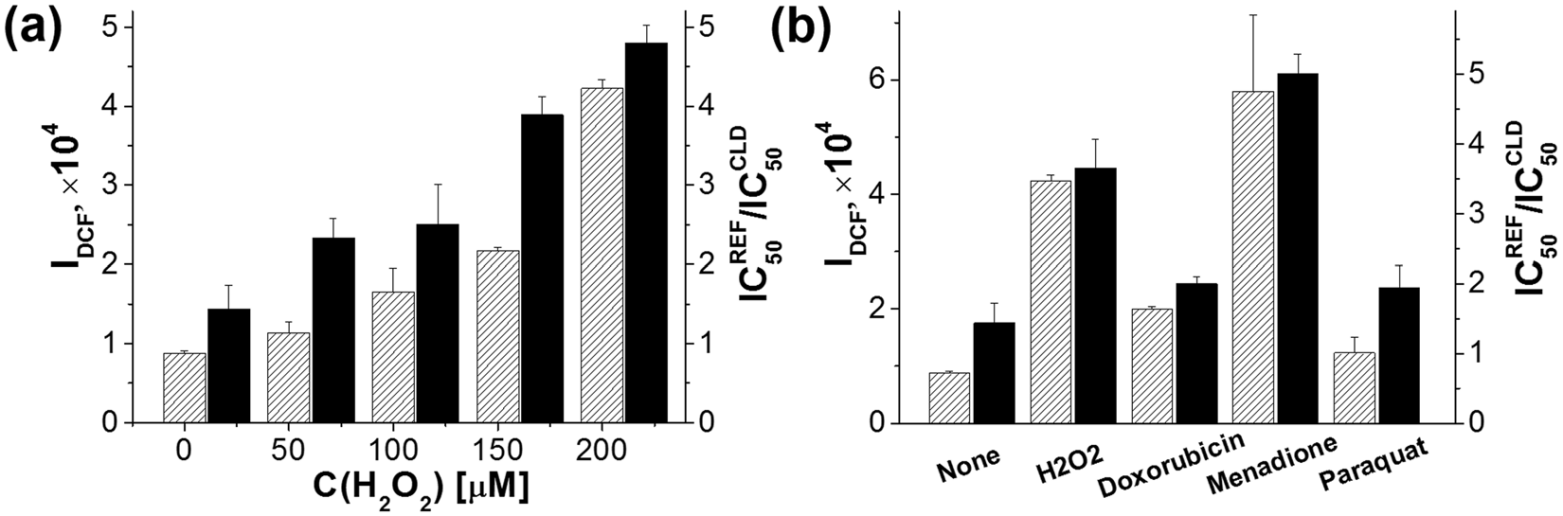
Correlation between CLD cytotoxicity and the level of oxidative stress. Effect of hydrogen peroxide (**a**) and other oxidative stress inducers (**b**) on DCF fluorescence intensity normalized to mg of cellular protein (shaded bars) and on the PS-mediated cytotoxicity of CLD expressed as $I{C}_{50}^{REF}/I{C}_{50}^{CLD}$ ratio (filled bars). CLD contained L64/DMP/POX/TMHP = 1:2.7:0.3:0.05 by weight.

Cell treatment with hydrogen peroxide in the concentration range up to 200 μM resulted in an increase of DCF fluorescence (Fig. 8a, shaded bars) indicating the induction of oxidative stress by H_2_O_2_. The relative PS-mediated cytotoxicity of CLD measured as $I{C}_{50}^{REF}/I{C}_{50}^{CLD}$ ratio increased in line with augmentation of H_2_O_2_ concentration (Fig. 8a, filled bars). This fact proves that the level of ROS determines the PS-mediated CLD cytotoxicity.

This conclusion was supported in the experiments with doxorubicin, menadione and paraquat, well-known inducers of oxidative stress^48–50^. All of them favoured ROS production as shown by the augment in DCF fluorescence (Fig. 8b, shaded bars) and enhanced the PS-mediated cytotoxicity of CLD (Fig. 8b, filled bars). A direct correlation between the relative increase in ROS production and cytotoxicity expressed as $I{C}_{50}^{REF}/I{C}_{50}^{CLD}$ values clearly show that the efficiency of PO-CL reaction in the dispersion captured by cells endowed their elimination.

## Discussion

Oxidative stress plays an important role in cancerogenesis^51, 52^ and proliferation of malignant cells^53–55^. As it has been deduced by López-Lázaro, this is due to the fundamental changes in cancer cells metabolism, which are expressed in the shift of the O_2_ metabolism “from the pathway that generates ATP to the pathway that generates ROS”^14^. Therefore prooxidant nanoparticles targeted to the cells with increased ROS production might be powerful for treatment of a broad spectrum of tumours irrespectively of their type. As hydrogen peroxide is the most stable representative of ROS family, it accumulates in the cells experiencing oxidative stress. We employed this peculiarity to eliminate tumour cells via PO-CL reaction between a synthetic polymeric oxalate and endogenous hydrogen peroxide.

The polymeric oxalate as a substrate of PO-CL reaction was synthesized via polycondensation of bisphenol A, oxalyl chloride, and oligomeric polypropylene oxide. The choice of building blocks for polyoxalate synthesis was guided by low hydrolytic stability of oxalic esters containing electron-withdrawing substituents in phenyl fragment^20, 56^. This consideration restricted our choice to biphenols with low acidities (high pK_a_ values). Our attempt to synthesize polyoxalate using 4,4′-dihydroxybiphenyl resulted in the copolymer practically insoluble in common solvents, pointing to the importance of flexibility of the biphenyl fragment. Therefore, we turned attention to bisphenol A containing aliphatic joint carbon between two hydroxyphenyl groups (Fig. 1) and therefore manifesting higher flexibility in comparison to 4,4’-dihydroxybiphenyl. In addition, bisphenol A exhibits prooxidant activity in the form of reactive phenoxyl-radicals when oxidized^57^. These properties favour biological activity of the resulting formulation. In this respect bisphenol A gains advantage over 4-hydroxybenzyl alcohol exerting antioxidant properties^17, 58^ and thus defending cells from the destructive action of singlet oxygen. For these reasons we have chosen bisphenol A for POX synthesis. Short oligo(propylene oxide) with M = 425 was used as an additive to further enhance solubility of the resulting product in organic solvents. Thus prepared polymer exhibited high solubility in DMP and THF.

The polymer was formulated into nanosized dispersions stabilized by Pluronic L64 as a biocompatible surfactant. Among the family of polymeric surfactants, Pluronic L64 exhibits relatively low cytotoxicity^23^ and high efficacy in solubilization and emulsification of aromatic substances^30^. It was reported to form stable xylene^59^ and tributylphospate^60^ emulsions. So, Pluronic L64 was selected for stabilization of POX dispersions in water solution.

DMP and THF are good solvents for aromatic oxalates according to Rauhut^10^. They both can be used for preparation of rather concentrated solutions of POX and display negligible cytotoxicity up to 0.4%. However, a striking difference in the properties of the dispersions prepared with these solvents was observed (Fig. 2d and S8a). It seems reasonable to suggest that this distinction results from different hydrophobicity of THF and DMP. Hydrophilic THF partitions from the particles into bulk water, while POX remaining in the micelles obviously appears to be exposed to hydrolysis. Therefore THF-based dispersions were inactive both in chemiluminescence and cytotoxicity measurements.

On the contrary, more hydrophobic DMP is poorly miscible with water and partitions into the hydrophobic core of Pluronic micelles resulting in the shift of its CMC (Fig. 3d). So, DMP forms hydrophobic core of the particles, preventing POX from hydrolysis within at least 2 h and the broad range of concentrations, even upon dilution to 0.03 mg/mL that was essential for cell experiments.

Colocalization of POX and TMHP dissolved in DMP favours efficiency of PO-CL reaction as follows from the fact that the PO-CL efficiency was reduced in excess of Pluronic L64 (see Fig. 4a and Supplementary Fig. S8b). The fact that the efficiency of peroxyoxalate chemiluminescence and the cytotoxicity both increased with the content of TMHP in the dispersions (see Fig. 4b and Supplementary Fig. S8d) proves our supposition that porphyrin fulfils the functions of an activator and a PS all in one. The resemblance of the dependences of the chemiluminescence efficiency and the PS-mediated cytotoxicity on the composition of the dispersions gives evidence that PO-CL reaction underlies toxic effects in cell culture.

We found that the obtained dispersions are efficient for production of singlet oxygen both in cell-free system (Fig. 4f) and in cells (Fig. 7). So, the designed approach can be applied for elimination of the cells which intensively produce ROS. Hydrogen peroxide is the most long-living and the less toxic form of ROS family^61^. Therefore, the elevation of the level of oxidative stress resulted in the increase in CLD cytotoxicity (Fig. 8a). This effect was independent on the way of induction of oxidative stress in the cells (Fig. 8b). All tested inducers of oxidative stress^52–54^ such as paraquat, the conventional antitumour drug doxorubicin, and even nutrient menadione were beneficial in PO-CL induced cytotoxicity. The latter is vitamin K3 and so can be regarded as safe for combined therapy. Combination with doxorubicin is of particular importance since it opens up the possibility to increase the efficiency of chemotherapy on the basis of conventional antitumour medications.

We are conscious that the approach reported in the present article does not pretend to be a design of a ready-to-use therapeutic formulation. However, we believe that further development of these systems will result in the appearance of novel anti-cancer treatment modalities capable in elimination of currently incurable tumours.

## Methods

### Synthesis of POX

The flame-dried reaction flask was charged with 10.0 mmol (1.268 g, 0.858 mL) and 10 mL of freshly distilled THF. The flask was immediately sealed and immersed into ice bath for 10 min. Then, the solution of the mixture of bisphenol A (2.057 g), PPO425 (0.412 g) and TEA (1.011 g, 1.389 mL) in 10 mL of THF was added dropwise. Additional 10–20 mL of THF were used to transfer the reagents quantitatively. The flask was kept in ice bath for additional half an hour and then the reaction was allowed to proceed at roo m temperature for 7 days. To isolate POX, the reaction mixture was passed through a Schott filter and the residue was repeatedly washed with freshly dried THF. The solvent was evaporated from the filtrate and the product was dispersed in anhydrous dioxane. The obtained suspension was centrifuged (12000 rpm, 15 min.), the supernatant was collected, and the pellet was washed twice with fresh portions of dioxane. Dioxane solutions were combined and the solvent was removed using rotary evaporator. Product yield varied from 50 to 62% in different experiments.

IR: 1750 cm^−1^ (C = O), 1176 cm^−1^ (C-O-C, ester and ether).

^1^H NMR: 1.67 ppm (m, 6 H, C(C**H**_3_)_2_); 7.2 and 6.7 ppm (m, 8 H, Ar); 1.14, 1.32 ppm (m, 3 H, -O-CH_2_-CH(C**H**_**3**_)-O-), 3.63 ppm (m, 3 H, -O-C**H**_**2**_-C**H**(CH_3_)-O-). 15% (mol.) Bisphenol A/PPO = 85/15 (mol.).

### Preparation of dispersions

Pluronic L64, POX/DMP solution and TMHP solution in acetonitrile were mixed thoroughly in a desirable weight ratio (the optimized composition L64/POX/DMP/TMHP was 1:2.7:0.3:0.05). Then appropriate amounts of PBS or DMEM preheated at 37 °C were added and the dispersion was shaken intensively using VibroFix VF-1 test tube shaker (IKA, Germany). Concentration of the dispersion is presented hereafter as the mass concentration of Pluronic L64 at a defined weight ratio of components.

### Chemiluminescent reaction

A sample of the dispersion was prepared in a thermostated quartz or optical glass cell (1 × 1 cm, 3.5 mL) and kept at 37 °С. Hydrogen peroxide and imidazole water solution (pH 7.4) were diluted with PBS and thermostated separately. The reaction was initiated by quick injection of H_2_O_2_+imidazole combined solution into the cell, the cover of the cell chamber being closed. The total volume of the sample was 2 mL. The chemiluminescent reaction was carried out at 37 °С in the thermostated cell holder. Chemiluminescence intensity was measured by Hitachi 650–10 S (Hitachi, Japan) spectrofluorimeter with photomultiplier tube R928 (Hamamatsu Photonics, Japan) with closed shutter of excited beam. Due to relatively slow reaction kinetics even in the presence of imidazole as a catalyst, the moment of 10-fold decrease in light intensity (I) as compared to maximum was considered to be the end of the reaction. Integral intensity (Q) was calculated for each experimental curve of light emission. This treatment of experimental curves corresponds to approximately 90% conversion. In all the experiments we used imidazole at concentration of 26 mM providing reaction duration of 20–30 minutes unless otherwise mentioned. Each experimental point is the average of at least 3 independent measurements.

### Induction of oxidative stress and evaluation of intracellular level of ROS

The cells were seeded in 24-well plates at a density about 9000/cm^2^ a day prior to the experiment. Oxidative stress inducers were added to the cells at a concentration close to the maximal tolerable concentration (MTC), i.e. the concentration ensuring viability of about 90% of the cells (C(H_2_O_2_) = 180 µM, C(Doxorubicin) = 6 µM, C(Menadione) = 20 µM, C(Paraquate) = 200 µM). The cells were incubated with oxidative stress inducers (hydrogen peroxide, doxorubicin, menadione or paraquate) in serum-free DMEM for 1 h at 37 °C. Then the solutions were replaced for the complete medium. Further processing of cell cultures was performed after additional 1 h incubation to ensure complete decomposition of exogenous hydrogen peroxide by serum catalase^38^.

To evaluate the level of oxidative stress, the cells were incubated with 20 μM of H_2_DCF-DA dissolved in indicator-free Hanks’ balanced salt solution for 30 min. The solution of H_2_DCF-DA was obtained by dilution of a freshly prepared 1.5 mM solution of this compound in water-free DMF. The cell monolayers were washed with PBS, collected by scrapping in 50 μL of PBS, transferred to the wells of black 96-well plates and the fluorescence was measured using Victor X5 2030 Multilabel Reader (Perkin Elmer, USA) at λ_ex_ = 490 nm, λ_em_ = 535 nm, exposition time 1.0 s/well. The obtained values were normalized to protein content which was measured after dissolution of the cells suspensions by addition of 0.1 mL of 1 N NaOH and further processing according to Lowry protocol^62^.

### LCSM

Confocal microscopy was performed using FluoView FV1000 microscope (Olympus Corp., Japan) equipped with spectral version scan unit and transmitted light detector. To observe TMHP fluorescence in the cells, the wavelength of excitation 405 nm (Diode laser) was used and the fluorescence was collected using an emission window set at 425–475 nm for acquisition of DAPI and 570–670 nm for TMHP fluorescence. Transmitted light Nomarski differential interference contrast (DIC) signal was detected simultaneously. Images were collected using the FV10 ASW 1.7 software (Olympus Corp., Japan).

To observe intracellular SOSG endoperoxide fluorescence, the cells were plated in glass bottom dishes at a density about 100,000 per sample a day before the experiment. One sample was kept untreated and the other samples were treated with H_2_O_2_ as specified above. After 1 h incubation of the cells in the presence of complete medium either complete CLD of reference dispersion without TMHP both containing 10 mM of SOSG were added to the cells and incubated for an additional hour. The control samples without dispersions were treated with 10 mM of SOSG in serum-free DMEM. All samples were prepared in DMEM without Phenol Red, sodium bicarbonate and fetal calf serum and supplemented with 10 mM HEPES. CLSM images were obtained from these samples at the scanning rate 20 μs/pixel. Each field was scanned no only once at excitation wavelength 488 nm (multiline Argon laser, the emission window 500–600 nm). All signals collected were adjusted to remain within the linear range of the detectors. The images were then treated by means of ImageJ 1.50i software (Wayne Rasband, National Institute of Health, USA) as follows. To measure mean pixel intensity, the cells were marked on microphotographs obtained via DIC channel and pixel intensity was measured on the fluorescence image. The data were averaged over about 300 cells on 10 images totally. Background on each image was averaged over 10 independent measurements and then the mean background value was subtracted from the mean value of cellular fluorescence in arbitrary fluorescence units.

### Evaluation of the dispersions cytotoxicity

The cells were seeded in 96-well plates at a density about 9000/cm^2^ a day before the experiment. 2 mL of serum-free DMEM were added to the mixture of 2 mg of Pluronic L64, 2.5 µL of 10% POX solution in DMP and 2–25 µL of TMHP solution in acetonitrile and thoroughly mixed with Vibrofix shaker. Thus prepared dispersions were sequentially diluted with serum-free DMEM and 100 µL of these solutions were added to the cells. After 1 h incubation at 37 °C the solutions were replaced for 100 µL of complete medium. Control samples contained no dispersions. The cells were allowed to grow for additional 3 days to reveal delayed toxic effects. The amount of living cells was determined by MTT test. 50 µL of 1 mg/mL methyltetrazolium blue solution in DMEM was added to the wells. 3 h later the medium was removed, formazan crystals were dissolved in 100 µL of DMSO, and the absorbance of the solutions at 550 nm was measured using Multiscan Plus (Titertek, USA) plate spectrophotometer. Cell survival was calculated as the mean fraction of survived cells in the test samples in relation to the mean control. All measurements were performed at least in triplicates. The cytotoxicity was evaluated as the concentration of the sample corresponding to the survival of 50% of cells (IC50). This value was estimated by non-linear fitting of the experimental data using Logistic function.

1 $$Survival, \% =\frac{100 \% }{1+{(\frac{IC50}{C})}^{p}},$$

where C - is the concentration of the sample and p - is the parameter indicating the steepness of the curve.

### Statistical analysis

Statistical analysis was performed according to t-test analysis using the Origin 7.5 software. Values of p < 0.05 were considered significant.

## Electronic supplementary material

Supplementary Information
