# Supplementary material for: Peroxyoxalate Chemiluminescent Reaction as a Tool for Elimination of Tumour Cells Under Oxidative Stress
Source: Sci Rep. 2017 Jun 13;7:3410. doi: 10.1038/s41598-017-03527-w (PMC5469817; doi:10.1038/s41598-017-03527-w)
Supplement: Supplementary file 1 — Supplementary Information [file 41598_2017_3527_MOESM1_ESM.doc]

**Supplementary INFORMATION to the article**

**DISPERSIONS CONTAINING POLYOXALATE ELIMINATE TUMOR CELLS UNDER OXIDATIVE STRESS CONDITIONS**

Andrey V. Romanyuk,^†^ Irina D. Grozdova,^†^ Alexander A. Ezhov,^‡^ Nickolay S. Melik-Nubarov*^,†^

^†^M.V. Lomonosov Moscow State University, School of Chemistry, GSP-1, Leninskie gory 1, build. 3, Moscow 119992, Russia

^‡^M.V. Lomonosov Moscow State University, Faculty of Physics, Leninskie gory 1, build. 2, Moscow 119991, Russia

**Synthesis and characterization of Polyoxalate**

Fig. S1**. Scheme of POX synthesis.**

**Fig. S2. GPC chromatogram trace for POX.** Molecular weight distribution of POX was determined by gel-permeation chromatography in THF as eluent at ambient temperature and at the flow rate 0.5 mL/min. The chromatograph was equipped with Styragel HR3 column (5 µm, 7.8 mm × 300 mm, Waters, USA), Altex 114M pump (USA), Waters 484 tunable absorbance detector (USA) and Smartline 2300 RI-detector (Knauer, Germany). The UV-detection was performed at 254 nm. A set of polystyrene standards (Sigma-Aldrich, USA) was used for calibration.


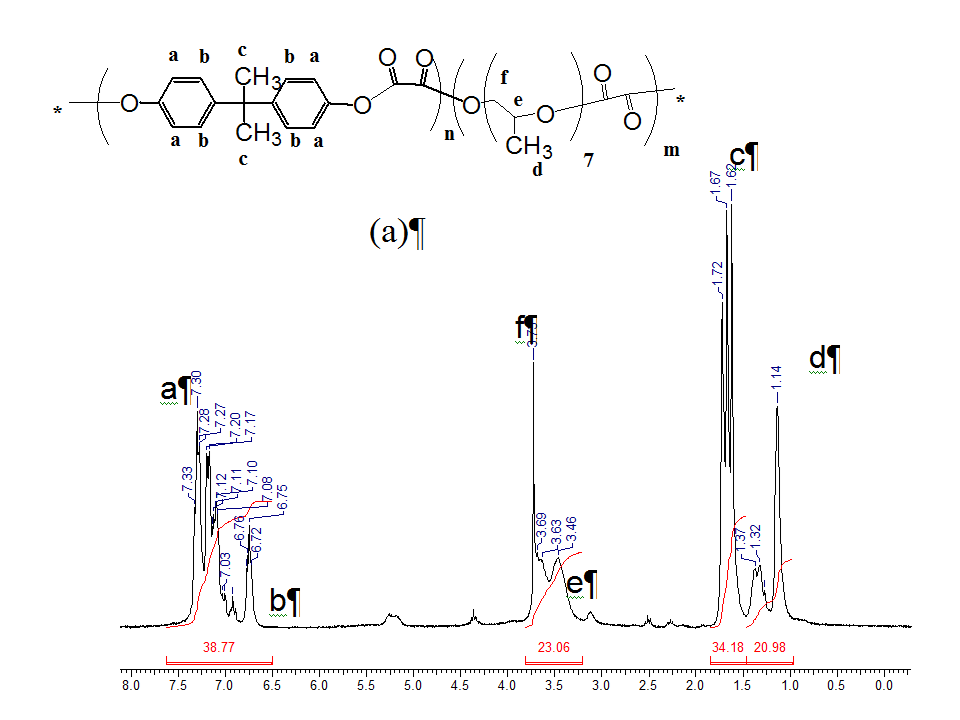


**Fig. S3. ^1^H NMR spectrum of POX.** ^1^H NMR spectra were recorded using spectrometer Bruker DRX500 (USA) with the instrument working frequency of 500 MHz. The frequencies of NMR shifts were calibrated using TMS as a standard and the residual signals of the protons from the solvents. The solutions of POX in CDCl_3_ (Deutero GmbH, Germany) were placed into standard ampoules. The spectra were obtained at room temperature.

**Estimation of the concentration range of existence of POX dispersions using 1,6-diphenyl-1,3,5-hexatriene solubilization technique**

Addition of DPH to the micelles of Pluronic L64 (Figure S4, curve1), the dispersion of DMP in Pluronic L64 (Figure S4, curve 2), the dispersion of POX/THF solution in Pluronic L64 (Figure S4, curve 3) and the dispersion of POX/DMP solution in Pluronic L64 (Figure S4, curve 4) at concentration of the surfactant about 1 mg/mL resulted in a considerable increase in DPH fluorescence owing to its partitioning into the hydrophophobic core of the dispersion particles. The sequential dilution of the dispersions at a constant concentration of the probe resulted in a gradual decrement of DPH fluorescence, however the critical concentration of the dispersions corresponding to the onset of hydrophobic phase was different. The data show that L64/POX/DMP dispersions retained at concentrations below 0.1 mg/mL.


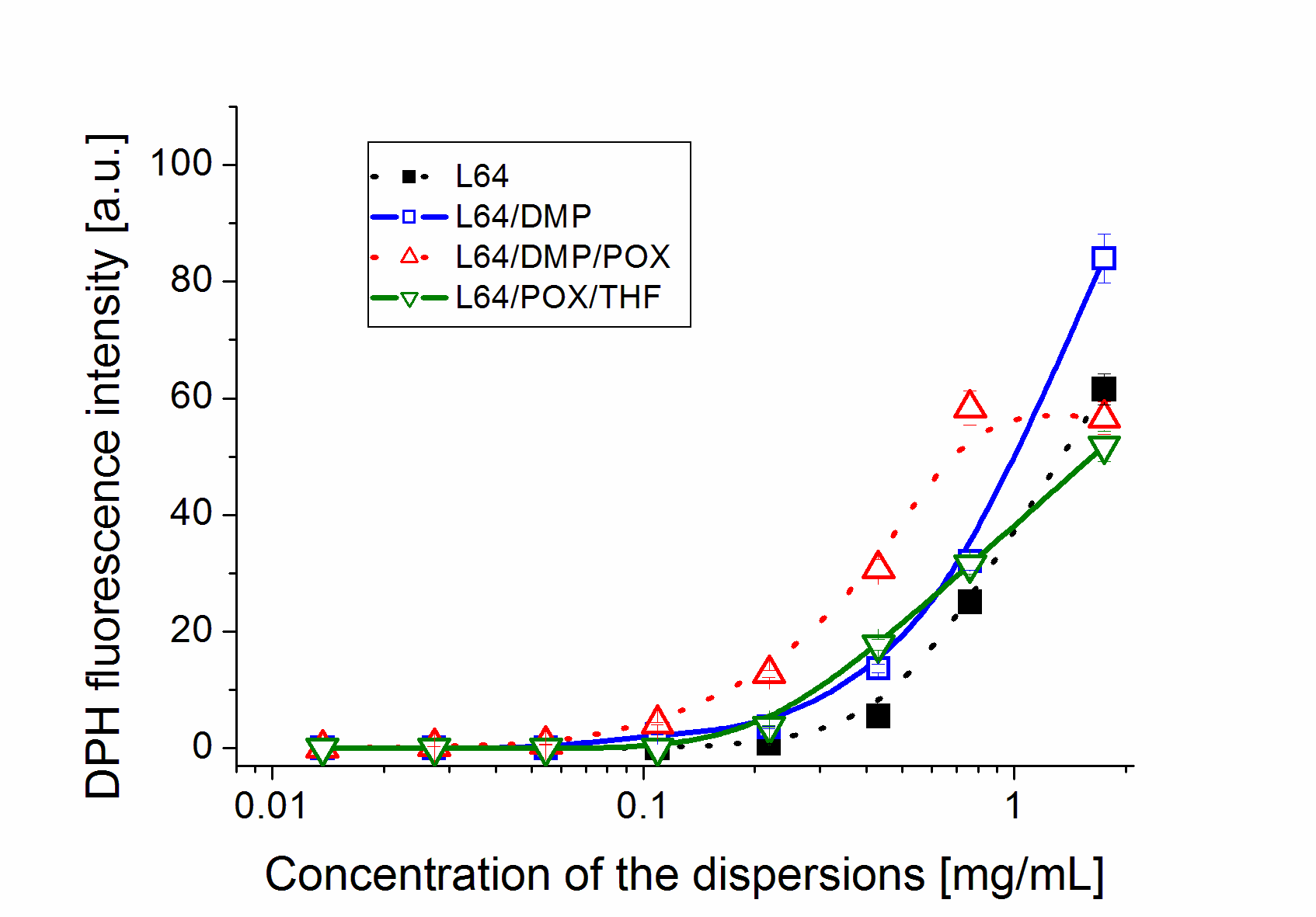


**Figure S4. Characterization of the POX-containing dispersions**. Dependence of DPH fluorescence intensity on the concentration of dispersions (1) L64, (2) L64/DMP (1:3 by mass), (3) L64/DMP/POX (1:2.7:0.3 by mass) and (4) L64/THF/POX (1:3.7:0.3 by mass) in PBS, 37^o^C. The concentration of dispersions is expressed in equivalent concentration of Pluronic L64.

**Kinetics of oxidation of Singlet Oxygen Sensor Green by hydrogen peroxide and its oxidation in the presence of CLD.**

**
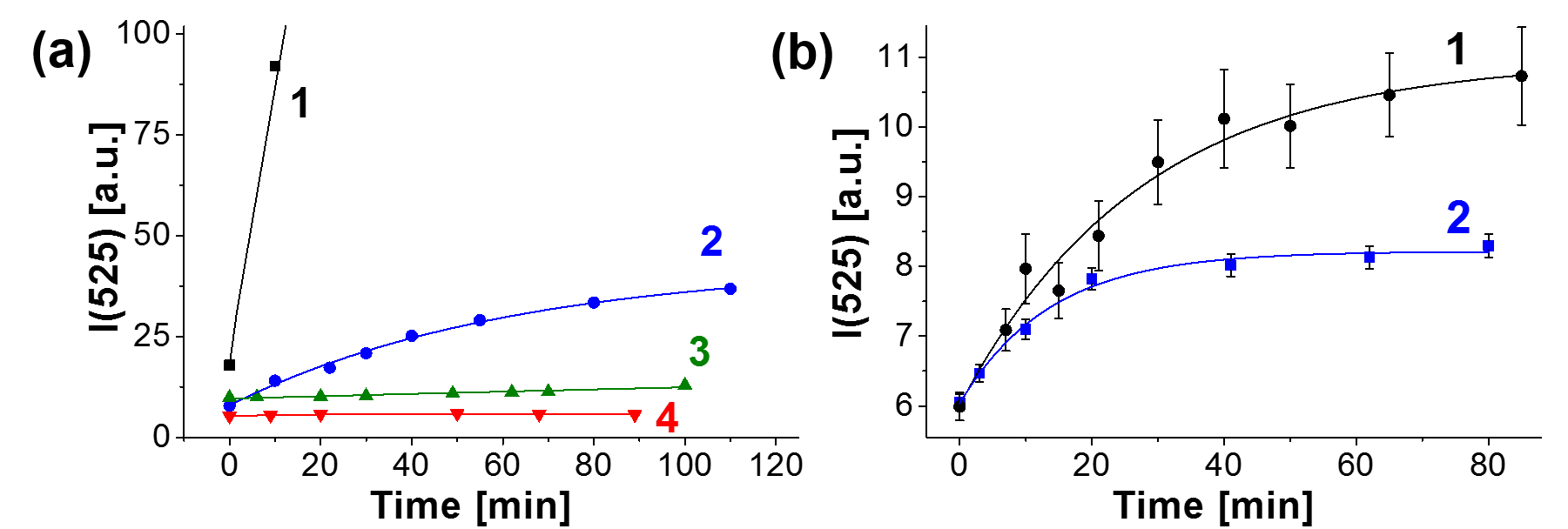
**

**Figure S5. Singlet oxygen generation during PO-CL reaction in the cell-free system.** (a) Kinetics of SOSG oxidation in the presence of 20 mM (1), 1 mM (2), 100 µM (3) and 30 µM (4) of H_2_O_2_. (b) Comparison of the kinetics of SOSG endoperoxide accumulation by virtue of PO-CL reaction between complete CLD and 30 µM H_2_O_2_ in PBS prepared in D_2_O (1) and H_2_O (2).

**Cytotoxicity of Hydrogen Peroxide for MCF-7/ADR cells.**

**Fig. S6. Cytotoxicity of hydrogen peroxide**. The cells (3,500/per well) were incubated with hydrogen peroxide for 1 h in serum-free medium and then were allowed to proliferate for additional 3 days. The survival rate was estimated using MTT test.

**Evaluation of the concentration of hydrogen peroxide in culture medium and changes in its concentration during exposure to complete**


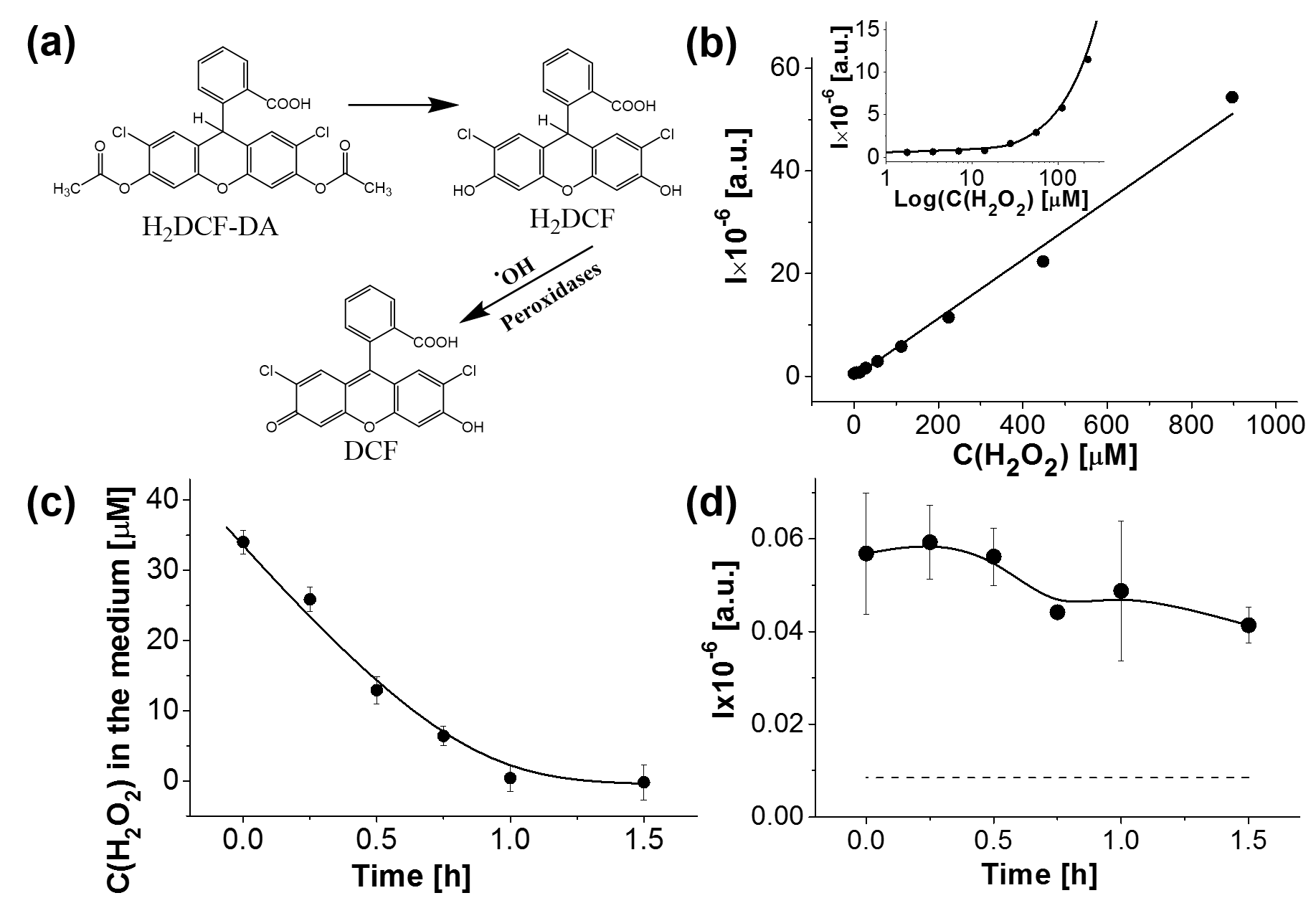


**Figure S7. Evaluation of the kinetics of H_2_O_2_ decay in culture medium upon exposure to fetal calf serum.** (a) Mechanism of 2’,7’-dichloro-dihydroflurescein diacetate oxidation in the cells under oxidative stress conditions according to ^1^. (b) The effect of H_2_O_2_ concentration on inducing DCF fluorescence in the presence of 0.2 µM of horseradish peroxidase in DMEM without Phenol Red and NaHCO_3_ and supplemented with 10 mM HEPES and 10% of fetal calf serum. The inset shows the detection limit of the assay. (c) Gradual decrease in H_2_O_2_ concentration in the medium during the exposure of the cells pretreated with 200 µM of hydrogen peroxide to complete medium containing 10% of foetal calf serum. (d) Changes in the intracellular DCF fluorescence during the exposure of cells to complete medium containing 10% of foetal calf serum. Dotted line shows DCF fluorescence in the intact cells.

**The effect of CLD composition on the PS-mediated cytotoxicity**

In contrast to CLD containing DMP, the POX/THF dispersions did not cause any PS-mediated cytotoxicity (cf. curves 1 and 2 in Figure S8a). This result is in full agreement with the previously demonstrated negligible efficiency of PO-CL reaction in L64/POX/THF dispersions in the cell-free system.

Cytotoxicity of CLD depended on L64/DMP mass ratio. $IC_{50}^{REF}/IC_{50}^{CLD}$ values were about 3-3.5 at the weight fraction of L64 0.17 and 0.34. Further increase in L64 content up to 0.68 resulted in an abrupt decrease in CLD mediated cytotoxicity (Figure S8b). These results are in conformity with the dependence of PO-CL reaction on Pluronic L64 concentration in the cell-free system (Figure 4a).

Complete accordance with the chemiluminescence efficiency was also observed while varying POX concentration in DMP. The increase in POX concentration in DMP from 5% to 10% resulted in 1.3-fold increase in $IC_{50}^{REF}/IC_{50}^{CLD}$value (Figure S8c) that is close to 1.5-fold elevation of the chemiluminescence efficiency observed for the same formulations in the cell-free systems (Figure 4b). Further increase in POX content did not lead to additional augmentation of the both cytotoxicity and chemiluminescence.

Finally, the $IC_{50}^{REF}/IC_{50}^{CLD}$ ratio increased with TMHP concentration, indicating that CLD cytotoxicity was mainly determined by the efficiency of singlet oxygen generation (Figure S8d). The result is also in conformity with the chemiluminescence measurements in the cell-free systems (Figure 4c). It means that TMHP in the designed CLD fulfilled the functions of the activator and PS.


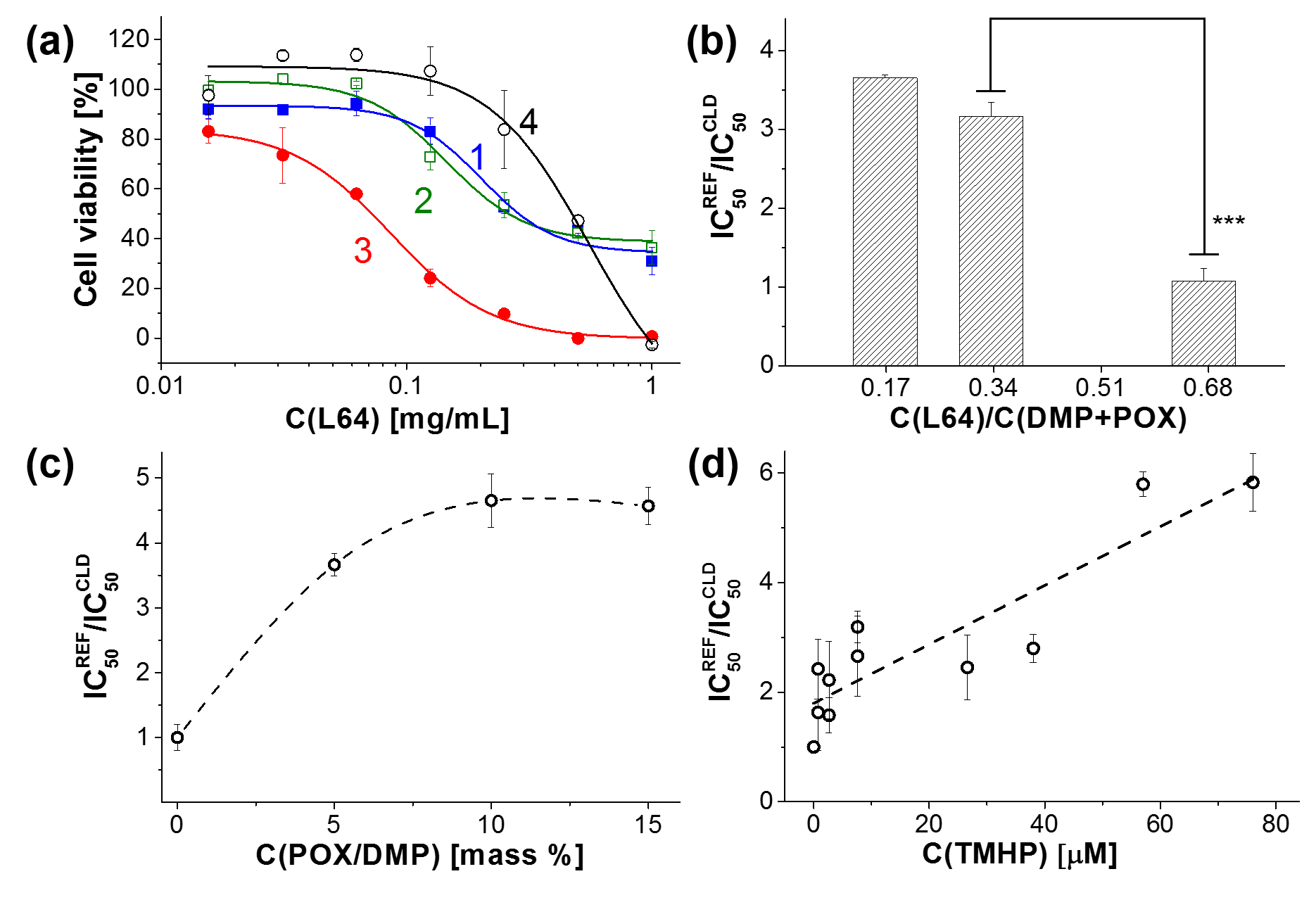


**Figure S8. Dependence of PS-mediated cytotoxicity on the composition of the dispersions.** (a) Comparison of the dispersions prepared from POX solutions in THF (curves 1, 2) and DMP (curves 3, 4). The cells were treated with complete (curves 1, 3) and reference formulations without TMHP (curves 2, 4). (b) Variation in L64/(DMP+POX) ratio at a constant (10% w/w) concentration of POX in DMP phase. (c) Variation in POX content at a constant (1:3) L64/DMP ratio. 7.6 μM TMHP was used in the experiments (a)-(c). (d) Effect of TMHP concentration in CLD at L64/DMP/POX weight ratio = 1:2.7:0.3. Oxidative stress in MCF-7/ADR cells was stimulated with 200 µM H_2_O_2_ in all experiments. Asterisks show significantly different bars according to t-test, p < 0.05.

**Methods**

**Dynamic light scattering (DLS).** The samples for DLS analysis were prepared immediately before the measurements by intensive shaking of the mixtures of Pluronic L64/DMP (1:3), Pluronic L64/ 10% POX solution in DMP (1:3 mass ratio) or Pluronic L64/5% POX solution in THF (1:4 mass ratio) after addition of dust-free PBS.

The autocorrelation functions and the average intensities of scattered light were measured by dynamic light scattering with goniometer PhotoCor (PhotoCor Corp., USA). Light was emitted by He-Ne-laser (λ = 633 nm, 15 mW). Autocorrelation functions of scattered light intensity fluctuation were collected by 288-channel correlator PhotoCor-SP with logarithmic time scale from 2.5 × 10^-8^ to 6800 s. The data were being accumulated for 10-15 minutes and autocorrelation functions were analyzed according to the method of regularization with the help of DynaLS software (PhotoCor Corp., United States) in order to obtain diffusion constant distribution of scattering particles. Values of hydrodynamic radii were calculated according to Stocks equation in approximation of spherical particles. All measurements were carried out at 37°С.

**Cell maintenance**. Multi-drug resistant epithelial-like adhesive human breast adenocarcinoma MCF7/ADR cells were a generous gift of Prof. A. A. Shtil from Russian Center of Oncology, Institute of Cancerogenesis (Moscow, Russia). The cells were maintained in DMEM containing 10% of foetal calf serum, 4 mM glutamine, 100 µg/ml streptomycin and 100 units/ml penicillin (further, complete medium). Cells were grown in the CO_2_-incubator (NAPCO, USA) at 37°C and 95% humidity in the atmosphere containing 5% CO_2_. The cells were routinely examined for mycoplasma and other DNA-containing organisms using DAPI according to the manufacturer’s instructions. Only uncontaminated cell culture was used in the experiments.

**Assay of extracellular hydrogen peroxide using 2’,7’-dichloro-dihydrofluorescein (H_2_DCF) and horseradish peroxidase.** The assay is based on the formation of highly fluorescent DCF from non-fluorescent H_2_DCF (Figure S6a) owing to oxidation by hydroxyl radicals produced from H_2_O_2_ by horseradish peroxidase^35,36^. To prepare deacetylted form of H_2_DCF-DA, 0.25 mL of 1 mM solution of the probe in methanol was mixed with 2 ml of 0.01M NaOH and incubated 20 min (r.t.). Then the solution was diluted with 10 mL of 25 mM phosphate buffer pH 7.4 to give colorless 50 μM solution H_2_DCF which became brightly yellow in about 10-12 h under atmospheric conditions. 50 μL of this solution were added into the wells of 96-wells black plate containing 100 μL of H_2_O_2_ solution in DMEM supplemented with 10% of foetal calf serum and 10 mM HEPES and deprived from Phenol Red and sodium bicarbonate. Finally 50 μL of 1 μM solution of horseradish peroxidase were added, the samples were incubated for about 15 min and DCF fluorescence was measured using Victor X5 2030 Multilabel Reader as described above**.**

**Measurements of chemiluminescence in cell monolayer.** 150-400 thousand of cells were seeded into 12-well plate a day before the experiment. The cells were treated with hydrogen peroxide as described above and incubated in the complete medium for 1.5 h to ensure complete decomposition of exogenous H_2_O_2_. 0.2 ml of the L64/POX/DMP/Perylen/Benzimidazole dispersions (1:2.7:0.3:0.12:0.11 weight ratio) prepared in serum-free DMEM without Phenol Red and sodium bicarbonate and supplemented with 10 mM HEPES were added into the wells. The plate was immediately placed into the holder of Victor X5 2030 Multilabel Reader preheated up to 37^o^C and chemiluminescence was measured at acquisition time 10 s. The aperture of the photomultiplier was adjusted to maximum. Measurement of the rate of light emission from each well was repeated each 5 min during about 1 h to obtain the kinetics of light emission. Integral light emission was calculated as a square under the curve. Actual amount of cells in the samples was calculated from the amount of protein determined in the wells after the completion of the experiment.

Calibration curves for the evaluation of the amount of H_2_O_2_ (Figure 6b in the manuscript) were obtained under the same conditions. To this end, twofold more concentrated dispersions were prepared in DMEM and mixed with equal volumes of H_2_O_2_ calibration solutions also prepared in DMEM. Measurement of the chemiluminescence was performed using the above described settings of the instrument.

**Literature cited**

1. Rota, C., Chignell, C. F. & Mason, R. P. Evidence for free radical formation during the oxidation of 2′-7′-dichlorofluo rescin to the fluorescent dye 2′-7′-dichlorofluorescein by horseradish peroxidase:Possible implications for oxidative stress measurements*. Free Radic. Biol. Me*d**. 2**7, 873–881 (1999).
